# The socioeconomic impact of infertility on women in developing countries

**Published:** 2016-03-28

**Authors:** M Patel

**Affiliations:** Groote Schuur Hospital, Cape Town, South Africa.

The World Health Organization (WHO) has classified infertility as a disease affecting approximately 15% of reproductive-aged couples. It is a disease that is not characterized by mortality but rather by the morbidity it inflicts on the individual and the couple. This morbidity includes social, economic, relationship and psychological aspects but is not confined to these. Women especially may be caught in a spiral of attempts to achieve their one social and evolutionary need namely to have a child.

A number of international decisions have placed infertility care in the context of reproductive health as a health priority. For many affected couples improvement or restoration of infertility-related reproductive health requires Assisted Reproductive Technology (ART). Although according to latest reports more than 1.6 million ART cycles were undertaken in 2008 (Chambers et al., 2013). The availability and accessibility of ART differs greatly between regions and countries. An important factor influencing accessibility is the cost of treatment and how these costs are covered. In many countries and especially in low resource settings, ART requires out-of-pocket payments by the consumer and while these may be affordable to some they may be impoverishingly expensive to others. The latter raises the question why couples would be willing to pay for treatment that they are unable to afford.

Studies from Africa and Asia have highlighted the many important roles of children which collectively allow their parents, especially their mothers, to become more esteemed members of the family and community (Dyer and Patel, 2012). Children are a reliable source of manpower in many rural and developing areas (Feldman-Savelsberg, 1994; Gerrits, 1997; Hollos, 2003; Barden-O’Fallen, 2005; Hollos et al., 2009) and provide economic security in old age; infertility often leads to instability in a marriage and the possibility of divorce or abandonment with consequent loss of financial security. Certain customary laws and cultural traditions lead to negative attitudes to infertile women and may potentiate the scourge of gender inequality.

Infertility may lead to abandonment and more economic hardship if women have to payback their bride wealth or pay bride wealth for husbands to enter into new unions (Nahar, 2012; Nahar et al., 2011). These social inequalities are reinforced when infertile women are treated as social servants by tending to the sick and infirm (Okonofua et al., 1997; Ruganga et al., 2001) or caring for the children of others (Okonofua et al., 1997; Dhont et al., 2011). Women may not be allowed to inherit or continue living in their husbands compound after he dies (Hollos, 2003; Hollos et al., 2009). Sons are seen to strengthen the lineage and the inheriting capacity of a family (Hollos, 2003) - consequently girl-children are seen as less important thereby reinforcing gender inequality.

The socioeconomic consequences of infertility are not easily overcome by availability to care. Cost of ART is an indicator of the underlying costliness of the countries health system. Consequently, it differs between countries, as does the out-of-pocket payment per individual couple. The cost to consumer is a function of the underlying cost of treatment, the level of subsidization or third party cost coverage and the available income of the consumer.

A study conducted in South Africa identified the extent of out-of-pocket-payment and financial coping mechanisms in couples accessing ART at a public, tertiary referral hospital where ART was subsidized but couples had to pay part of the expenditure (Dyer et al., 2013). Participants were divided into tertiles of socioeconomic status based on annual household per capita expenditure. Catastrophic expenditure was defined as an out-of-pocket payment greater than 40% of the annual non-food household expenditure. According to results, 20% of couples incurred catastrophic expenditure - 51% of couples were in the lowest socioeconomic tertile. Financial coping strategies included reduced expenditure on food and clothing, use of savings and borrowing money that incurred interest. Half the couples had to take on extra work to offset the cost of ART.

Not only does the cost and funding models of treatment provide an important explanation to the differences in the utilization of available treatment, but they also help to explain clinical practices especially relating to embryo transfer.

Some countries like Israel and Australia have state funded programmes which enable certain state-imposed restrictions to be placed on these cycles. Examples of these include age limits and the requirement for single embryo transfer thereby decreasing the downstream indirect cost of multiple pregnancies and prematurity associated with this (Chambers et al., 2013).

However, developing countries do not have an authority to standardize cost. This leaves women vulnerable to exploitation by both western and traditional medical practitioners (Sundby, 1997, Okonofua et al., 1997). Efforts to make ART affordable in developing countries have been undertaken by non-profit organizations such as the Walking Egg and the Low-Cost IVF Foundation.

Methods to reduce cost of ART exist and must be pursued wherever possible. Introduction of third party funding usually requires the imposition of some restrictions or regulations. Restrictions may apply as to who is given access to ART while regulations may apply regarding number of embryos transferred with the view if reducing the biggest risk of ART, and its resultant downstream costs, namely that of multiple pregnancies. Additional cost reducing strategies include less aggressive stimulation cycles with less monitoring, novel use of incubation techniques, earlier embryo transfers and effective use of cryopreservation. There should be specific start and end points to treatment modalities with age-appropriate and cause-appropriate interventions. Clinics may offer risk sharing, package pricing for multiple cycles or cross subsidization.

Although it is assumed that lower cost of treatment will improve access, this is not always the case. Some studies have indicated that lower socioeconomic and certain ethnic groups may still be disadvantaged (Chambers, 2013). Care must also be taken that more affordable treatment does not lead to inappropriate perpetuation of ART in some couples are caught in an unrelenting pursuit for a child.

The benefits of ART are difficult to quantify but important. They centre on quality of life and happiness. The majority of people consider parenthood as part of the fulfilment of life goals. Cost utility analysis is the main method governments use to guide allocations of public resources to specific health outcomes. This is usually measured in quality adjusted life years (QALY’s), which captures improvement in health among living patients. It is very difficult to quantify this for fertility treatment as the creation of new life cannot be captured in the indicator. However, United Kingdom National Institute of Clinical Excellence fertility guidelines incorporated QALY’s and concluded that under most clinically appropriate circumstances access to ART treatment and single embryo transfer represented good value for money from a societal perspective (www.guidanceniceorg.uk).

Improving access to infertility care requires a two-faceted approach. Infertile couples must be able to access quality care at affordable cost, however this is attained. In addition, efforts to prevent infertility should be escalated - according to the WHO up to 45% of adult conditions develop during adolescence and this is the target group for education regarding preventative strategies.
